# Effects of different social experiences on emotional state in mice

**DOI:** 10.1038/s41598-020-71994-9

**Published:** 2020-09-17

**Authors:** Viktoria Krakenberg, Sophie Siestrup, Rupert Palme, Sylvia Kaiser, Norbert Sachser, S. Helene Richter

**Affiliations:** 1 Department of Behavioural Biology, University of Münster, Badestraße 13, 48149 Münster, Germany; 2 Otto Creutzfeldt Center for Cognitive and Behavioral Neuroscience, University of Münster, Münster, Germany; 3 Department of Biomedical Sciences, University of Veterinary Medicine, Vienna, Austria; 4 Present Address: Department of Psychology, University of Münster, Münster, Germany

**Keywords:** Ecology, Zoology

## Abstract

A comprehensive understanding of animals’ emotions can be achieved by combining cognitive, behavioural, and physiological measures. Applying such a multi-method approach, we here examined the emotional state of mice after they had made one of three different social experiences: either a mildly “adverse”, a “beneficial”, or a “neutral” experience. Using a recently established touchscreen paradigm, cognitive judgement bias was assessed twice, once before and once after the respective experience. Anxiety-like behaviour was examined using a standardised battery of behavioural tests and faecal corticosterone metabolite concentrations were measured. Surprisingly, only minor effects of the social experiences on the animals’ cognitive judgement bias and no effects on anxiety-like behaviour and corticosterone metabolite levels were found. It might be speculated that the experiences provided were not strong enough to exert the expected impact on the animals’ emotional state. Alternatively, the intensive training procedure necessary for cognitive judgement bias testing might have had a cognitive enrichment effect, potentially countering external influences. While further investigations are required to ascertain the specific causes underlying our findings, the present study adds essential empirical data to the so far scarce amount of studies combining cognitive, behavioural, and physiological measures of emotional state in mice.

## Introduction

The assessment of emotional states in non-human animals (hereafter: animals) is of major importance for multiple research fields, including for example animal welfare science, psychopharmacology and behavioural neuroscience^1,2^. However, finding valid and objective measures of animals’ emotions can be challenging^3^. In practice, scientists traditionally rely on physiological as well as behavioural indicators of affective state, often used alongside each other. Physiological indicators commonly include parameters related to the study of stress, for instance heart rate or stress hormone concentrations^4^. While these reliably reflect arousal states, they are considered to be unsuitable for discriminating between states of differential emotional valence^4,5^. The additional assessment of behavioural parameters facilitates a more comprehensive understanding of animals’ emotional states. For instance, facial and vocal expressions as well as spontaneous behaviours (e.g. approach and avoidance or play behaviour) can be assessed, allowing for the interpretation of emotional valence^4^. Adding to this, standardised behavioural test batteries are commonly applied to assess fear and anxiety-like behaviour, especially in disciplines like neuroscience or psychopharmacology^4,6^.

Over the last fifteen years, a novel approach has gained increasing importance, targeting the cognitive component of emotion via so called cognitive biases^4,6–8^. The cognitive bias concept derives from human psychology and is based on the phenomenon that emotions can influence cognitive processes^9^. For example, individuals in a positive affective state tend to interpret ambiguous stimuli in a more “optimistic” way compared to individuals in a negative state^9,10^. This so-called cognitive judgement bias can serve as a proxy measure of the valence of affective states, also in animals^4,6,8^. In a seminal study, Harding and colleagues introduced an experimental paradigm to systematically assess cognitive judgement bias in rats^7^. Inspired by their work, judgement bias tests have been developed for a multitude of different species^11–15^. The majority of studies across species reports mood-congruent judgement biases. Thus, animals in a negative (e.g. anxiety-like) state generally display “pessimistic” judgement biases, animals in a positive affective state (e.g. induced via environmental enrichment) “optimistic” ones^16–19^ (but see also^20–22^).

Although mice are the predominantly used mammalian animal model^23,24^, only little is known about factors associated with variations in judgement bias in this species. So far, stereotypic behaviour, considered to reflect a negative affective state, has been linked to differences in judgement bias^25,26^. Furthermore, studies investigating different strains of mice indicate the potential involvement of a genetic component^14,25,27^ (but see also^28^). Most interestingly for the framework of this study, stressful experiences have been discussed as potential modulators of judgement bias in mice, yet again, evidence remains unclear^26^. Thus, the modulation of judgement biases in mice is far from being understood. Moreover, the focus has been put on the investigation of negative affective states, while effects of putatively positive experiences remain understudied (but see also^29^).

In the present study, we therefore aimed to investigate the influence of both a positive and a negative affect manipulation on the cognitive judgement bias of male laboratory mice. In contrast to previous studies that have used rather artificial treatments, we aimed to provide treatments of high ecological relevance. For this purpose, social experiences of sexual as well as agonistic nature were chosen. As a mildly “adverse” experience, one group of animals was repeatedly confronted with a dominant male opponent. Losing such an aggressive confrontation has been shown to increase anxiety-like behaviour in rodents^30^, and a study in rats even revealed an influence of social defeat on judgement bias^17^. As a putatively “beneficial” experience, we presented another group of mice with freshly collected female urine. The presentation of female urinary pheromones can induce positive affect in male mice, as it reduces anxiety-like behaviour^31^ and aggression^32^. Sniffing female urine has further been shown to trigger male ultrasonic courtship vocalisations^33^ which are suggested to reflect positive affect^34,35^.

We assessed cognitive judgement bias twice, once before and once after mice had made the respective social experience, using a recently implemented touchscreen paradigm^36^. We expected a mood-congruent shift in judgement bias after the experience phase. To cover not only cognitive, but also physiological and behavioural measures of emotional states, we additionally assessed faecal corticosterone metabolite concentrations reflecting hypothalamic-pituitary-adrenal axis activity and anxiety-like behaviour in a battery of standardised tests. With this multi-method approach we intended to gain a comprehensive picture of the impact of different social experiences on the emotional state of mice.

## Animals and methods

### Animals and housing conditions

The present study was conducted with 24 male C57BL/6J mice, purchased from a professional breeder (Charles River Laboratories, Research Models and Services, Germany GmbH, Sulzfeld, Germany) at the age of five weeks. Upon arrival, mice were housed in same-sex groups of 3 individuals per cage (Makrolon cages type III, 38 × 23 × 15 cm^3^), since in sub-adult male mice, the occurrence of escalated aggression is very unlikely. However, with the males becoming adult, the probability of escalated agonistic encounters increases. Therefore, at the age of nine weeks, mice were transferred to single housing conditions to avoid any escalated aggressive interactions. Please note that the question whether to house male laboratory mice singly or in groups is under ongoing discussion and there is still no “gold standard” regarding its solution. For current discussions about recommendations for male mouse housing see^37,38^. Cages were equipped with wood chips as bedding material (TierWohl Super, J. Rettenmaier & Söhne GmbH + Co.KG, Rosenberg, Germany), a wooden stick, a semi-transparent red plastic house (11.1 × 11.1 × 5.5 cm^3^, Tecniplast Deutschland GmbH, Hohenpeißenberg, Germany), and a paper tissue. Housing rooms were maintained at a reversed 12 h dark/light cycle with lights off at 8 a.m., a temperature of approximately 23 °C, and a relative humidity of about 50%. The animals had ad libitum access to water and food (Altromin 1324, Altromin Spezialfutter GmbH & Co. KG, Lage, Germany) until the beginning of the touchscreen training phase. From then on they were mildly food restricted to 90–95% of their ad libitum feeding weights in order to enhance their motivation to work for food rewards. As neither distinct negative effects of such a restricted feeding protocol^39^, nor an interference with judgement bias assessment^17,18^ could be detected in previous studies, we considered this method to not affect the emotional state of the mice itself. Weights were monitored on a daily basis using a digital scale (weighing capacity: 150 g, resolution: 0.1 g; CM 150-1 N, Kern, Ballingen, Germany).

In addition to the experimental animals, 16 group-housed adult female C57BL/6J mice and 5 single-housed adult male NMRI mice, purchased from Charles River Laboratories, were used to provide the test animals with social experiences.

### Ethics statement

All procedures complied with the regulations covering animal experimentation within Germany (Animal Welfare Act), the EU (European Communities Council DIRECTIVE 2010/63/EU), and the fundamental principles of the Basel Declaration, and were approved by the local (Gesundheits- und Veterinäramt Münster, Nordrhein-Westfalen) and federal authorities (Landesamt für Natur, Umwelt und Verbraucherschutz Nordrhein-Westfalen “LANUV NRW”, reference number 84-02.04.2015.A441).

### Experimental design

**Figure 1:**
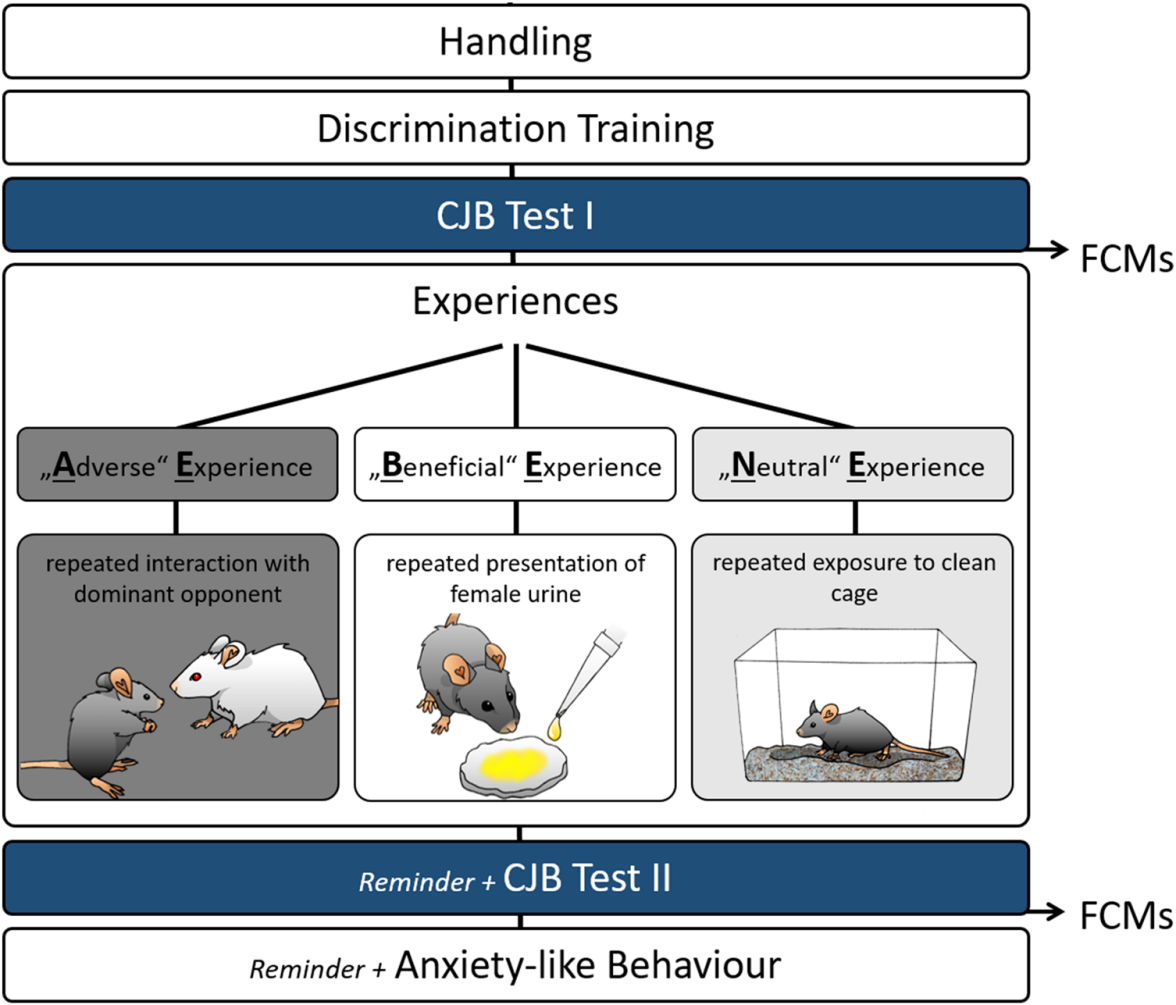
Experimental design. Mice were habituated to cup handling before they underwent daily training sessions until successful acquisition of the discrimination task. Afterwards, they were tested in the cognitive judgement bias (CJB) test. During the following phase, mice repeatedly made one out of three different experiences: mildly “adverse”, “beneficial”, or “neutral”. They were then tested for their CJB again. During this second test phase, a so-called reminder was presented before each test session with the aim to re-evoke the affective state the animals experienced during the treatment phase. On the last day of each CJB test phase, faecal corticosterone metabolite concentrations (FCMs) were assessed. Subsequently, animals were tested for anxiety-like behaviour. Again, they were presented with reminders before each behavioural test.

In this study, the effects of different social experiences on important correlates of animal emotions, comprising cognitive (judgement bias), behavioural (anxiety-like and exploratory behaviour) as well as physiological (stress hormone levels) measures, were investigated. The experiment comprised six phases: a handling phase, a training phase, a first cognitive judgement bias (CJB) test phase, an experience phase, a second CJB test phase, and a behavioural test phase (Fig. 1).

During the handling phase starting at PND 69, mice were first habituated to cup handling for 5 days and thereafter underwent daily training sessions to learn the discrimination task required for CJB testing, starting at PND 76. Afterwards, the animals’ initial CJB was assessed (start test phase 1: PND 223 ± 77; for details on CJB training and testing see following section).

During a subsequent experience phase starting at PND 230 ± 77, mice were exposed to one of three different experiences, each comprising three group-specific encounters, classified as either mildly “adverse”, “beneficial”, or “neutral”. Encounters took place under red light between 2:45 p.m. and 4:35 p.m. on 3 different days, always separated by a gap day. The mildly “adverse experience” group (AE group, n = 8) repeatedly encountered a dominant opponent of the aggressive NMRI strain^40^, with each confrontation lasting maximally 10 minutes^30,41^. Confrontations were terminated in cases of high aggression. The “beneficial experience” group (BE group, n = 8) was repeatedly presented with freshly collected urine of an unfamiliar C57BL/6J female for 10 minutes^31^. To provide all subjects with comparable experiences, we controlled for the females’ oestrus state. Since the time of oestrus in mice is relatively short^42^, urine from non-oestrous females was used in order to keep the total number of involved females low. The “neutral experience” group (NE group, n = 8) served as a control group and was repeatedly placed into a novel cage containing clean bedding material for 10 min.

Following the experience phase, CJB was assessed again to investigate the influence of the respective experience on the animals’ judgement bias (start test phase 2: PND 237 ± 77). In this second test phase, a so-called *reminder* was presented immediately before each test session. These *reminders* were introduced to acutely re-evoke the affective state the mice experienced during the encounters of the treatment phase. *Reminders* took place immediately before each test session of the second CJB test phase. For this purpose, mice were placed into a cage (Makrolon type II cage; 22 × 16 × 14 cm^3^) filled with bedding for 3 min. For AE mice, another 25 ml of soiled bedding from the home cage of the last NMRI male encountered were added. For BE mice, the same was done with soiled bedding from the home cage of the last female of which urine had been presented.

On the last day of each CJB test phase, faeces samples were obtained to assess corticosterone metabolite (FCM) concentrations. Finally, animals underwent a battery of standard behavioural tests for anxiety-like behaviour and exploratory locomotion (elevated plus maze test (EPM), dark-light test (DL), and open field test (OF); start: PND 245 ± 77). Before each test session, a *reminder* was presented again.

The allocation of mice to the treatment groups was pseudo-randomised, so that balanced numbers of mice with different learning speeds were present in each group. The testing order of mice was randomised once before the first CJB test and subsequently maintained for the following CJB and behavioural test sessions. As *reminders* were provided immediately before CJB testing as well as before the subsequent behavioural tests, blinding of the experimenter was not possible.

### The touchscreen-based cognitive judgement bias test

#### Procedure

The same apparatus as described previously was used^28,36^ (Bussey-Saksida Mouse Touch Screen Chambers, Model 80614, Campden Instruments Ltd., Loughborough, Leics., UK). Mice underwent daily touchscreen sessions at intervals of approximately 24 h on maximally 6 consecutive days. Before each session, each mouse was taken out of its home cage and weighed. In a red semi-transparent box (21 × 21 × 15 cm^3^) the animal was then transported to a separate room where it was placed into the touchscreen chamber. The session was started and ended after a maximum number of trials had been performed or after a training step-specific duration. All touchscreen sessions were conducted during the dark phase between 8.15 a.m. and 1 p.m.

#### Paradigm

**Figure 2:**
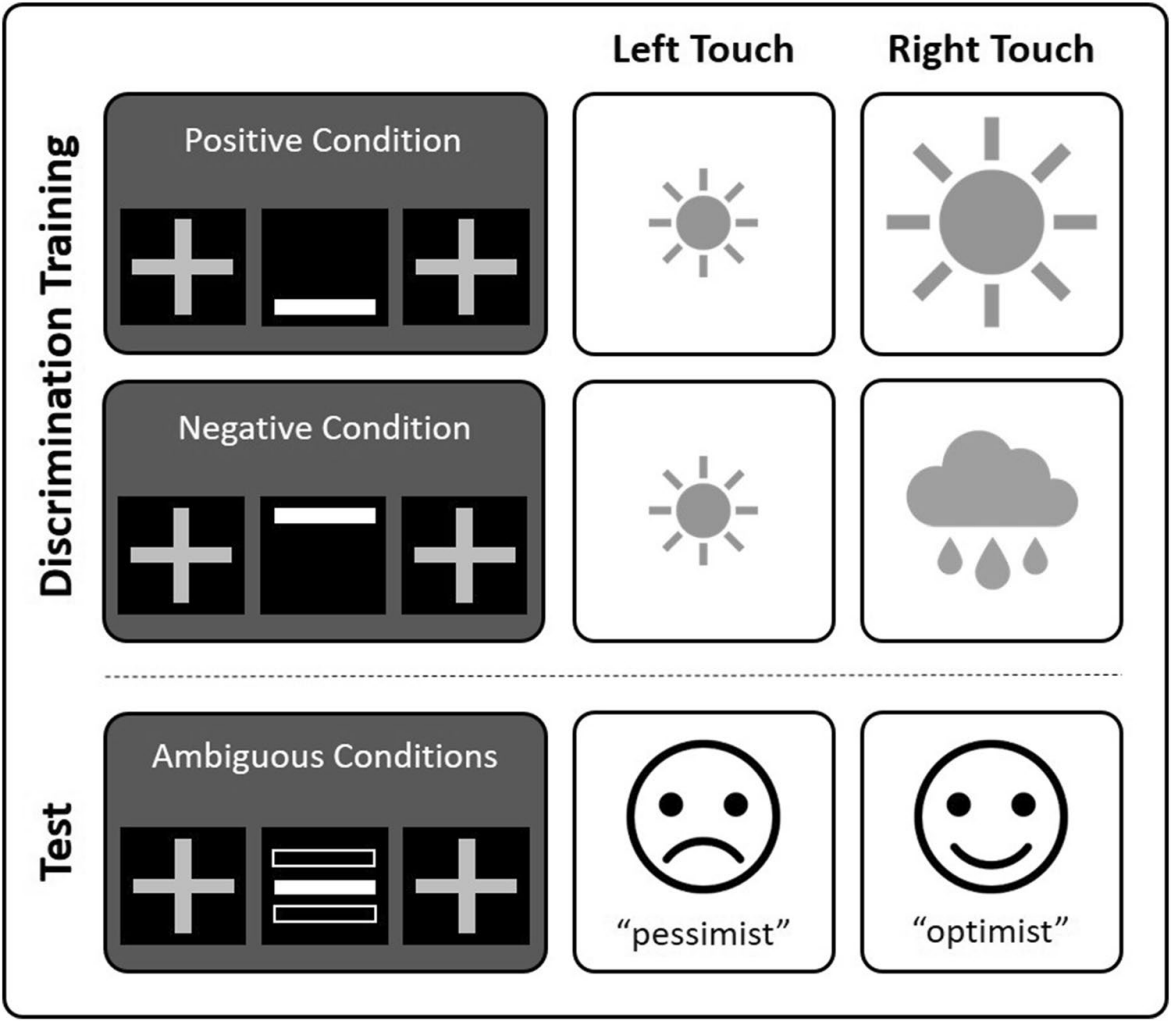
Touchscreen-based cognitive bias paradigm. Mice were trained to distinguish between bars displayed at the top (negative condition) or bottom (positive condition) of a central field of a touchscreen. In this example, mice learned to touch right for a large reward during the positive condition and to touch left for a small reward during the negative condition (the association between positive/negative cue and the correct touch side was counterbalanced across mice). During the test, mice were presented with cues displayed at three intermediate positions: near positive, middle and near negative. The relative number of “optimistic”-like responses to these ambiguous conditions served as outcome measures of the animals’ cognitive judgement bias. Figure adopted from Krakenberg et al. (2019) with permission from Elsevier^36^.

The paradigm applied here was the same as described previously with minor modifications^36^. Briefly, mice were trained to distinguish between a positive and a negative condition (Fig. 2). The positive condition was signalled by a bar at the bottom (5 cm below upper edge) of the cue-presentation field, the negative condition by a bar at the top (1 cm below upper edge). Mice had to touch either the left or right touch field in response to the cues. A correct touch in the positive condition led to the delivery of a large reward (12 μl of sweet condensed milk, diluted 1:4 in tap water, in the following “SCM”). An incorrect touch resulted in the delivery of a small reward (4 μl of SCM). In the negative condition, correct touches led to the delivery of a small reward (4 μl of SCM), while incorrect touches resulted in a mild “punishment” (5 s *time out* and *houselight* on). Mice had to learn to touch the high-rewarded side in the positive condition and the small-rewarded side in the negative condition. The small-rewarded touch field was the same in both conditions. The association between condition and correct touch side was the same for each individual but counterbalanced between mice. For a detailed description of the training procedure please see the supplementary material. After successful training, animals underwent CJB testing. The two cognitive bias test phases took place on five consecutive days each. During each CJB test session, three types of ambiguous cues, interspersed with the learned reference cues, were presented. These were bars at three intermediate positions: near positive (NP, 4 cm below upper edge), middle (M, 3 cm below upper edge) and near negative (NN, 2 cm below upper edge). Touches in response to these ambiguous cues resulted in a neutral outcome (neither a reward nor a “punishment”). The animals’ judgements made in response to these cues indicated whether they interpreted them according to the positive (“optimistic” response) or negative (“pessimistic” response) reference cue, serving as a measure of CJB.

Each test session comprised 54 trials. Per session, each type of ambiguous cue was presented twice, interspersed with 48 training trials. Per test phase, each mouse was presented with each ambiguous cue ten times and each trained cue 120 times.

#### Behavioural measures

Responses to ambiguous cues served as a measure of the animals’ CJB. Touches according to the positive condition were defined as “optimistic” choices, touches according to the negative condition were defined as “pessimistic” choices. Out of all responses per condition, a “choice score” was calculated as previously^28,36^ according to the following formula:

$$ Choice\,Score = \frac{{N\,choices ( {\text{``}}optimistic{\text{''}} ) - N\,choices ({\text{``}}pessimistic{\text{''}})}}{ N\,choices ({\text{``}}optimistic{\text{''}} + {\text{``}}pessimistic{\text{''}})} $$

The choice score could range between − 1 to + 1. Higher scores indicated a higher proportion of “optimistic” choices and consequently a relatively positive CJB compared to lower scores. Please note that choice scores are not an absolute, but a relative measure of CJB and that the term was chosen for the sake of intuitiveness.

### Anxiety-like behaviour and exploratory locomotion

Mice were tested in three tests on anxiety-like behaviour and exploratory locomotion in the following order: the elevated plus-maze test (EPM), the dark-light test (DL) and the open field test (OF). The sequence of tests followed recommendations to schedule tests that are more sensitive to previous experience at the beginning of such a battery, and to conduct potentially more stressful tests towards the end^43,44^. Tests were carried out at intervals of at least 48 h and were performed in a room different from the housing room between 12:45 p.m. and 3:35 p.m. Test equipment was cleaned with 70% ethanol between subjects. Behaviour was recorded with a webcam (Logitech Webcam Pro 9000) and the animals’ movements during the EPM and OF were automatically analysed by the video tracking system ANY-maze (ANY-maze version 4.99, Stoelting Co., Wood Dale, IL, USA). Videos of the DL were analysed manually by an experienced observer (Sophie Siestrup). For apparatus descriptions and details about testing procedures see supplementary material.

### Faecal corticosterone metabolites

The basal levels of adrenocortical activity of the subjects were monitored non-invasively by measuring faecal corticosterone metabolites^45–47^ (FCMs). Faeces samples of each individual were collected on the last day of the first CJB test week (= before the experience phase) and on the last day of the second CJB test week (= after the experience phase). During the dark phase, a peak of FCMs can be found in the faeces 4–6 h after the exposure to a stressor^45^. For this reason, faeces samples were collected 5.5–8.5 h after an individual finished CJB testing to ensure that faeces collection could be finished in the dark phase. For sample collection, mice were placed in Makrolon cages type III with a thin layer of bedding material and clean enrichment items as present in the home cage. Water was available ad libitum. After the sampling period of 3 h, mice were transferred to novel clean cages together with the enrichment items. All faeces produced during this time were collected and frozen at − 20 °C. Faecal samples were dried and homogenised and aliquots of 0.05 g were extracted with 1 ml of 80% methanol. Samples were then analysed using a 5α-pregnane-3β, 11β, 21-triol-20-one enzyme immunoassay (for details see^45,46^). Intra- and inter-assay coefficients of variation were < 10% and < 12%, respectively.

### Data analysis

To check for the assumptions of parametric analysis, residuals of all data were analysed for heteroscedasticity and normal distribution graphically and using the Shapiro-Wilk normality test. If the assumptions were not met, data were transformed whenever possible (DL: latency to enter light compartment, logarithmic transformation). As CJB test data did not meet the assumptions of parametric analysis even after transformation, untransformed data were analysed using non-parametric tests.

Data from behavioural tests were analysed using a linear mixed effect model (LMM) with “experience” as fixed factor and “age” as random factor, followed by Holm-Bonferroni post hoc comparisons. Faecal corticosterone metabolite data were analysed using an LMM with “experience” and “time” as fixed factors, and “age” and “individual” as random factors.

In order to examine whether mice interpreted the conditions of the CJB test differently, data were pooled across animals for each condition and each test phase and analysed using the Friedman test. Post hoc comparisons between conditions were conducted using the Holm-Bonferroni-corrected Wilcoxon signed-rank test. The Wilcoxon signed-rank test was also used for within-group comparisons of choice scores before and after the experience phase. The Kruskal-Wallis test was used for between-group comparisons of choice scores. Subsequent post hoc comparisons were carried out using the Holm-Bonferroni-corrected Wilcoxon rank-sum test (unpaired).

Differences were considered significant at *p* ≤ 0.05. Whenever LMMs were used, effect sizes were calculated additionally to p-values as partial eta squared (η^2^_*p*_). Statistical analyses were performed using the software R^48^ (www.r-project.org, open source). Graphs were created using the software SigmaPlot for Windows (Version 12.5, Build 12.5.0.38, Systat Software, Inc. 2011).

## Results

### Cognitive judgement bias

**Figure 3:**
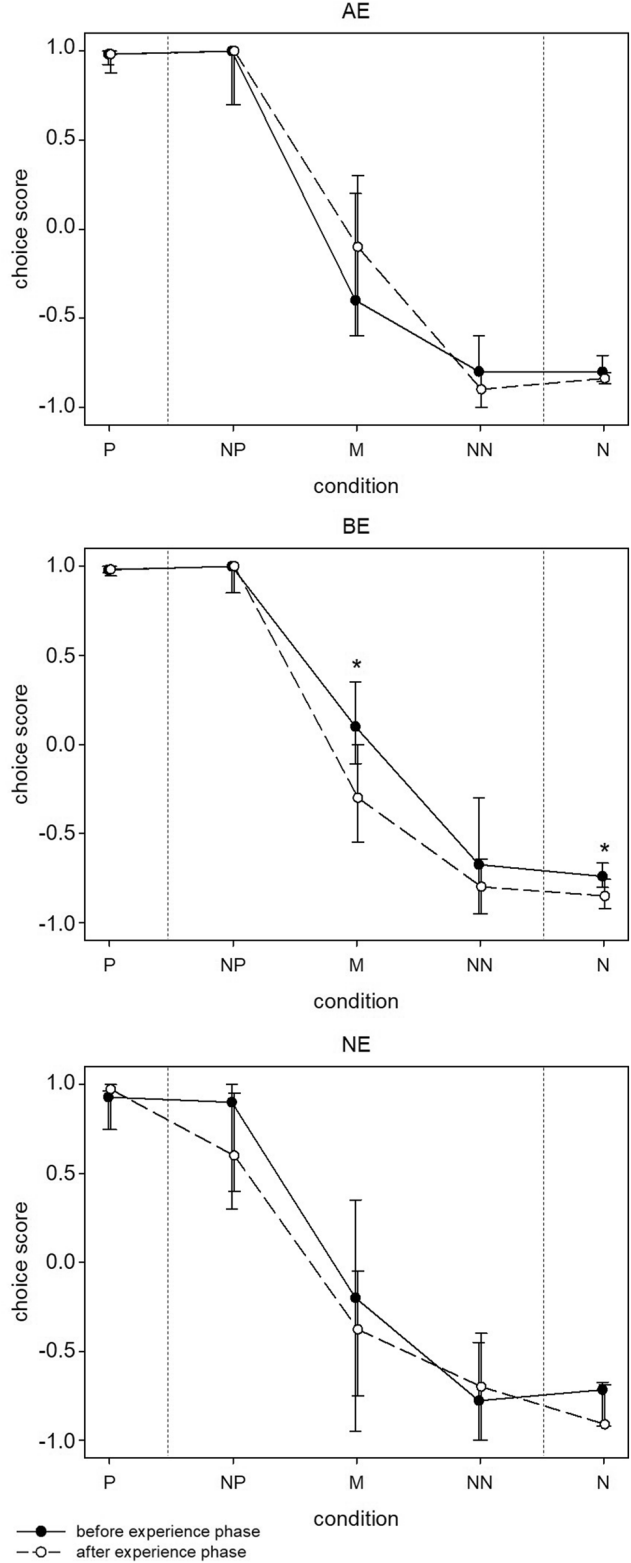
Cognitive judgement bias. Choice scores of AE, BE and NE mice in response to the three ambiguous conditions before and after the experience phase, presented as medians and 25th and 75th percentiles. AE: mildly “adverse” experience, BE: “beneficial” experience, NE: “neutral” experience. P: positive, NP: near positive, M: middle, NN: near negative, N: negative. Statistics: Wilcoxon signed-rank test, n_AE_ = n_BE_ = n_NE_ = 8. *p ≤ 0.05.

During both cognitive judgement bias test phases, mice interpreted the five conditions significantly differently as revealed by the analysis of choice scores pooled across groups (Friedman test, before experience phase: χ^2^_(4)_ = 80.1, *p* < 0.001, after experience phase: χ^2^_(4)_ = 6.88, *p* < 0.001; for post hoc comparisons see supplementary Fig. 1 and supplementary Table 2). Descriptively, choice scores of each group of mice resulted in response curves with highest scores in the positive and near positive condition, lowest in the near negative and negative condition, and intermediate scores in the middle condition (Fig. 3).

In order to detect potential shifts in the animals’ choice scores in response to the experiences, scores before and after the experience phase were compared within each group of mice using the Wilcoxon signed-rank test (for statistical parameters of all within-group comparisons see Table 1). In both the AE and NE group, no differences between choice scores before and after the experience phase could be detected in any of the five conditions. Solely BE mice displayed significantly lower choice scores in the middle as well as in the negative condition after the treatment phase (Wilcoxon signed-rank test, middle condition: V = 33, *p* = 0.04, negative condition: V = 32, *p* = 0.05).

**Table 1:** Statistical analysis of cognitive judgement bias test data.

Condition	Within-group comparisons:						Between-group comparisons			
	*Wilcoxon signed-rank test*						*Kruskal–Wallis test*			
	AE		BE		NE		Before		After	
	V	p	V	p	V	p	Χ^2^	p	Χ^2^	p
P	13.0	0.68	15.0	0.40	13.0	0.93	5.55	***0.06***	0.41	0.82
NP	2.5	1.00	4.0	0.85	8.5	0.89	1.99	0.37	6.88	**0.03**
M	9.5	0.50	33.0	**0.04**	14.0	0.53	1.92	0.38	0.97	0.62
NN	28.0	0.18	20.5	0.31	4.5	0.50	0.47	0.79	2.07	0.36
N	22.0	0.64	32.0	**0.05**	25.0	0.38	1.43	0.49	1.14	0.57

AE: mildly “adverse” experience, BE: “beneficial” experience, NE: “neutral” experience. P: positive, NP: near positive, M: middle, NN: near negative, N: negative. Statistical information given: V and p-value for Wilcoxon signed-rank test, Χ^2^ and p-value for Kruskal-Wallis test. Sample sizes: n_AE_ = n_BE_ = n_NE_ = 8. Bold: *p* ≤ 0.05, bold italic: 0.05 < *p* < 0.1.

To detect potential differences between the three treatment groups, choice scores in response to each condition were compared between AE, BE and NE mice using the Kruskal–Wallis test (for statistical parameters of all between-group comparisons see Table 1). Before the experience phase, there was a trend for a difference in choice scores between the three groups in the positive condition (Kruskal-Wallis test, χ^2^_(2)_ = 5.55, *p* = 0.06). Descriptively, NE mice displayed lower scores compared to AE and BE mice, however, no statistically significant pairwise differences could be detected based on post hoc comparisons (Wilcoxon rank-sum test, AE vs. BE: W = 36.5, *p* = 0.66; AE vs. NE: W = 46.5, *p* = 0.14; BE vs. NE: W = 54, *p* = 0.02; please note that using the Holm-Bonferroni correction for three pairwise comparisons the smallest of the 3 p-values has to be ≤ 0.017 for an effect to be significant at the 0.05 level). Regarding all remaining conditions, we did not detect any significant differences between the three groups before the experience phase. After the experience phase, a significant difference could be detected within the near positive condition (Kruskal-Wallis test, χ^2^_(2)_ = 6.88, *p* = 0.03). Descriptively, NE mice displayed lower choice scores compared to both other groups, however, no significant pairwise differences could be detected based on post hoc comparisons (Wilcoxon rank-sum test, AE vs. BE: W = 32, *p* = 0.84, AE vs. NE: W = 15, *p* = 0.06, BE vs. NE: W = 11, *p* = 0.02; please note that using the Holm-Bonferroni correction for three pairwise comparisons the smallest of the 3 p-values has to be ≤ 0.017 for an effect to be significant at the 0.05 level). Regarding all remaining conditions, again, no significant differences between the three groups were detected.

### Anxiety-like and exploratory behaviour

Anxiety-like and exploratory behaviour were assessed using the elevated plus-maze test (EPM), dark light test (DL) and open field test (OF). Table 2 gives an overview of the statistical parameters of the analysis.

**Table 2:** Statistical analysis of anxiety-like and exploratory behaviour.

Parameter		AE	BE	NE	LMM			
		Mean ± SEM	Mean ± SEM	Mean ± SEM	F	p	η^2^_*p*_	Transf
**Elevated plus maze test**								
Time spent on open arms (%)	A	38.0 ± 5.2	32.6 ± 2.9	32.9 ± 4.8	0.27	0.77	0.03	NT
Entries into open arms (%)	A	40.4 ± 3.5	38.7 ± 2.8	40.1 ± 2.9	0.08	0.93	0.01	NT
Distance travelled on open arms (m)	A	2.5 ± 0.3	2.3 ± 0.2	1.7 ± 0.3	2.60	0.10	0.21	NT
Sum of entries (#)	L	21.1 ± 1.4	24.0 ± 0.8	23.3 ± 2.4	0.69	0.51	0.06	NT
**Dark light test**								
Latency to enter light compartment (s)	A	4.1 ± 1.0	5.2 ± 1.3	7.6 ± 3.1	0.20	0.82	0.02	Log
Time spent in light compartment (s)	A	136.3 ± 16.4	147.8 ± 9.3	139.7 ± 10.6	0.28	0.77	0.03	NT
Entries into light compartment (#)	L	14.1 ± 1.1	13.8 ± 0.7	17.8 ± 1.4	3.73	**0.04**	0.30	NT
**Open field test**								
Time spent in centre (s)	A	13.7 ± 2.0	20.1 ± 2.6	14.6 ± 1.5	2.47	0.11	0.19	NT
Entries into centre (#)	A	7.6 ± 1.1	10.8 ± 1.0	7.9 ± 0.9	2.67	0.10	0.23	NT
Distance travelled (m)	L	32.1 ± 1.7	34.3 ± 1.0	40.3 ± 4.0	2.96	0.08	0.26	NT

Data are presented as untransformed means for the three groups (AE, BE, NE) ± standard error of the means (SEM). Statistical information given: main effects of experience (LMM: F-ratio, p-value, η^2^_p_) and transformation used for the statistical analysis (NT = not transformed, Log = logarithmic transformation). Sample sizes: n_AE_ = n_BE_ = n_NE_ = 8. A = anxiety-like behaviour, L = exploratory locomotion, bold = *p* ≤ 0.05.

**Figure 4:**
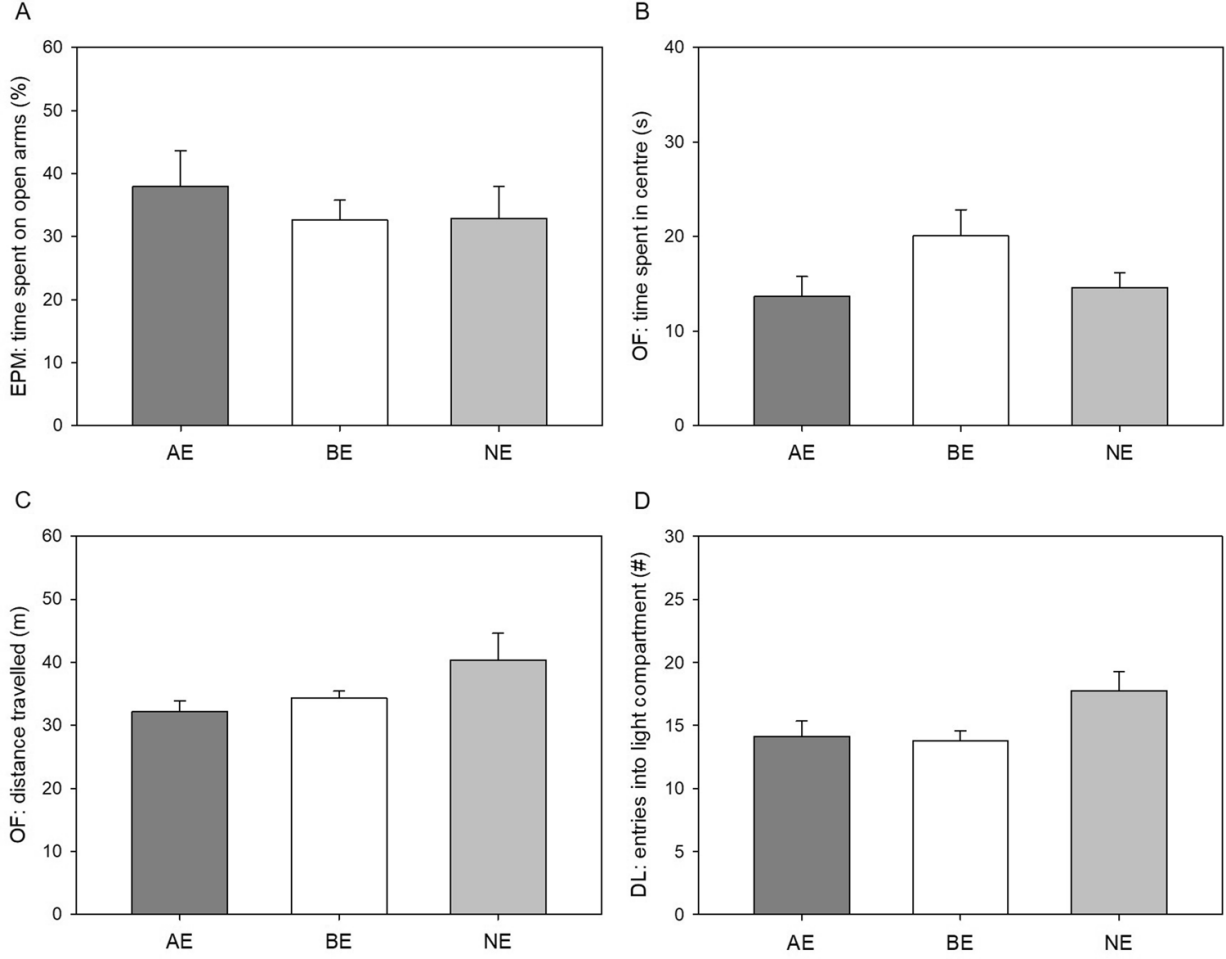
Anxiety-like behaviour and exploratory locomotion. Data are presented as means ± SEM. AE: mildly “adverse” experience, BE: “beneficial” experience, NE: “neutral” experience. (**A**) Time spent on open arms in elevated plus maze test (EPM), (**B**) Time spent in centre in open field test (OF), (**C**) Distance travelled in open field test (OF), (**D**) Entries into light compartment in dark light test (DL). Statistics: LMM with Holm-Bonferroni post hoc comparisons, n_AE_ = n_BE_ = n_NE_ = 8.

We did not detect significant main effects of experience on the parameters reflecting anxiety-like behaviour in the EPM, DL, and OF (for statistical details see Table 2, Fig. 4). Similarly, no significant main effects of experience on the parameters reflecting exploratory locomotion could be detected in the EPM and OF (for statistical details see Table 2, Fig. 4). However, in the DL, there was a significant main effect of experience on the number of entries the mice made into the light compartment of the apparatus (F_(2,17.81)_ = 3.73, *p* = 0.04, η^2^_*p*_ = 0.23; Fig. 4D). Descriptively, NE mice entered the light compartment more often than AE and BE mice, but pairwise differences were not statistically significant (Holm-Bonferroni post hoc comparison, NE vs. AE: *p* = 0.09; NE vs. BE: *p* = 0.08; AE vs. BE: *p* = 0.84).

### Faecal corticosterone metabolite concentrations

**Figure 5:**
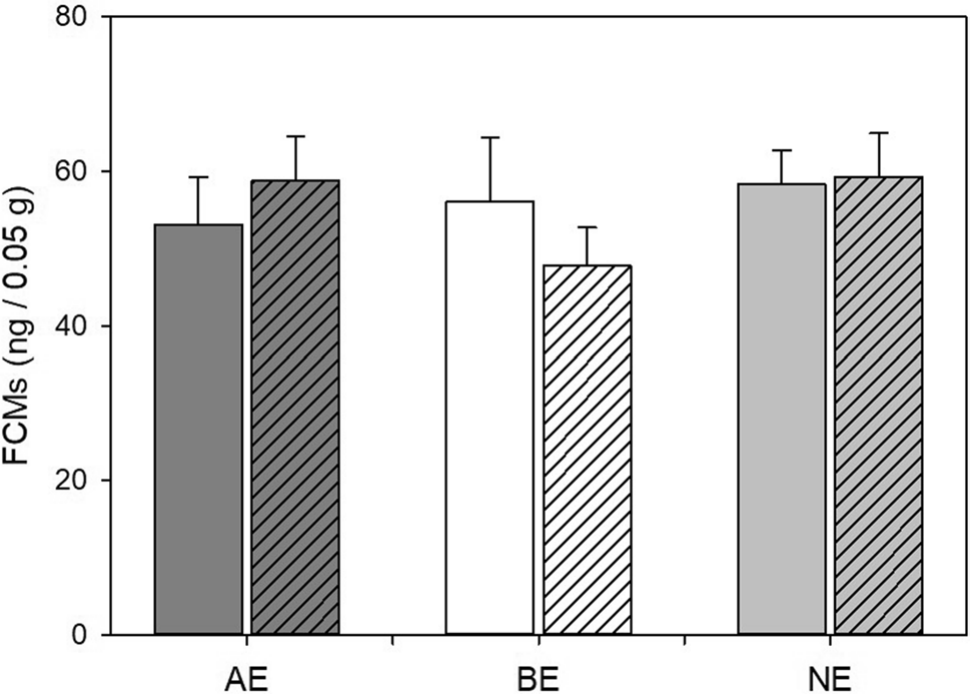
Faecal corticosterone metabolites (FCMs). Data are presented as means ± SEM. AE: mildly “adverse” experience, BE: “beneficial” experience, NE: “neutral” experience. Plain bars: before experience phase, striped bars: after experience phase. Statistics: LMM with Holm-Bonferroni post hoc comparisons, sample sizes before experience phase: n_AE_ = n_NE_ = 7, n_BE_ = 8; sample sizes after experience phase: n_AE_ = n_BE_ = n_NE_ = 8.

We neither detected a significant main effect of experience (F_(2,18.78)_ = 0.4, *p* = 0.72, η^2^_*p*_ = 0.04) nor of time point (F_(1,20.17)_ = 0.06, *p* = 0.81, η^2^_*p*_ < 0.01) on corticosterone metabolite concentrations. Likewise, no significant experience x time interaction could be found (LMM, F_(2,20.15)_ = 1.33, *p* = 0.29, η^2^_*p*_ = 0.12; Fig. 5).

## Discussion

Combining physiological, behavioural and cognitive correlates of emotional states is currently considered to be the most promising way to comprehensively assess emotional states of animals^2,4^. Applying such a multi-method approach, we here examined the effects of a putatively mildly “adverse” and a putatively “beneficial” experience on the emotional state of mice. Overall, only minor effects of the experiences on the animals’ choice scores and no effects on their anxiety-like behaviour and faecal corticosterone metabolite concentrations were found.

In the cognitive bias test, choice scores in response to the five conditions resulted in a curve that is typical for judgement bias tests across species e.g.^14^. This result is consistent with previous studies and confirms the general applicability of the touchscreen-based cognitive judgement bias paradigm^14,28,36,49^. Furthermore, no significant between-group differences were found. Likewise, no significant differences between choice scores before and after the experience phase could be detected in AE and NE mice. Yet, in BE mice, choice scores towards the middle condition significantly decreased after the experience phase, hinting at a pessimistic-like shift in judgement bias. However, we also found a significant decrease in the choice scores of this group in the negative condition, revealing a general negative shift of the animals’ response curve. This suggests that the animals’ choices in response to the ambiguous conditions do not solely reflect their judgement bias, but may additionally be influenced by other factors, such as learning accuracy or perceived reward value^26,50^. Consequently, the difference in choice scores towards the middle condition found in BE mice should be interpreted with caution. Thus, in summary, we did not detect clear effects of the different social experiences on the animals’ cognitive judgement bias in the present study.

While equivocal findings are not an exception in the field of cognitive bias research in mice^26,29^, the present results still deviate from our expectations based on previous studies, reporting effects of the same experiences as provided here on the emotional state of mice^30,31,41^. Interestingly, however, we also did not detect effects of the social experiences on anxiety-like behaviour, exploratory locomotion and corticosterone metabolite levels. Thus, not only cognitive, but also behavioural and endocrine proxy measures of emotional state obtained in this study point into a similar direction.

In search of a reasonable explanation for these findings, the social experiences provided require closer consideration. Regarding the efficacy of the putatively “beneficial” social experience, i.e. the repeated presentation of female urine, the oestrus state of the females might have influenced the results. Here, we provided urine of non-oestrous females. However, urine from females in oestrus, or even the direct contact with an oestrous female, might have enhanced the efficacy of the treatment due to a higher ecological relevance for the subjects.

Concerning the mildly “adverse” experience, we here provided three confrontations with a dominant male opponent. This experience was chosen since it has been shown to lead to increased levels of anxiety-like behaviour, lower levels of exploratory locomotion^30^ and an elevation of faecal corticosterone metabolite concentrations^41^ in previous studies in mice. Yet, the here applied procedure differs from that of a study in rats: Papciak and colleagues^17^ applied chronic social defeat in form of daily confrontations over the course of three weeks which caused a negative shift in judgement bias. In comparison to such a chronic stress paradigm, the here applied “adverse” experience was comparably milder, and therefore potentially less effective at inducing a negative emotional state. Thus, it would be interesting to investigate potentially more effective emotion manipulating treatments within future studies.

Despite a reduced efficacy of the experiences, however, there could also be another, alternative explanation for the findings of this study: a potential influence of the intensive touchscreen training phase which is required as a prerequisite for the cognitive judgement bias test. Indeed, the use of touchscreen paradigms for rodents, as well as discrimination training alone, have been proposed to act as cognitive enrichment^51,52^. This assumption finds recent support by a study conducted in our lab. Heterozygous serotonin transporter knockout mice showed a decrease in anxiety-like behaviour after cognitive bias testing using the touchscreen method, suggesting a beneficial influence of this procedure^28^. Moreover, it has been argued that enrichment-like properties of training procedures can potentially mask the influence of other, especially negative, experiences^8,51,53^. Therefore, touchscreen training in the present study might have had a positive influence on the animals’ emotional state, and thus might have buffered the impact of the social experiences, particularly the mildly “adverse” social confrontations. Arguing in favour of this hypothesis, it could incidentally be observed by the experimenter that AE mice showed offensive aggressive behaviours during confrontations with an opponent, something that has rarely been observed previously during social defeat paradigms. Yet, this novel hypothesis remains to be thoroughly investigated in the future, especially considering the use of appropriate control groups.

In summary, the present study adds essential empirical data to the so far scarce amount of studies investigating the effects of ecologically relevant emotion manipulating treatments on a set of cognitive, behavioural, and physiological measures of emotional state in mice. Since no clear effects of the treatments could be detected here, further research in this field is required to elucidate the specific effects of the applied experiences, as well as the applicability of the cognitive judgement bias paradigm. Furthermore, the present findings led to a novel hypothesis: touchscreen training might exert a pronounced and presumably positive effect on the animals’ emotional state. This assumption deserves closer attention in future studies and is currently under systematic investigation in our lab.

## Supplementary information

Supplementary Information 1.

Supplementary Information 2.

Supplementary Information 3.
