# Supplementary material for: Effects of different social experiences on emotional state in mice
Source: Sci Rep. 2020 Sep 17;10:15255. doi: 10.1038/s41598-020-71994-9 (PMC7498458; doi:10.1038/s41598-020-71994-9)
Supplement: Supplementary file 3 — Supplementary Information 3. [file 41598_2020_71994_MOESM3_ESM.docx]

**Effects of different social experiences on emotional state in mice**

Viktoria Krakenberg^a,b,+,^*, Sophie Siestrup^a,b,c,+^, Rupert Palme^d^, Sylvia Kaiser^a,b^, Norbert Sachser^a,b^, S. Helene Richter^a,b^

^a^Department of Behavioural Biology, University of Münster, Germany

^b^Otto Creutzfeldt Center for Cognitive and Behavioral Neuroscience, University of Münster, Germany

^c^Present address: Department of Psychology, University of Münster, Germany

^d^Department of Biomedical Sciences, University of Veterinary Medicine, Vienna, Austria

^+^Equal contribution

*Corresponding author: viktoria.krakenberg@uni-muenster.de

**Supplementary methods**

**Touchscreen Pre-Training**

Before mice were confronted with the discrimination task, they had to acquire basic skills to operate within the touchscreen chambers during the so called *pre-training*^1,2,3^, starting at PND 76. The skills to be learned included touching for rewards, associating reward delivery with a tone and initiating trials by nose poking into the reward magazine. Mice were also accustomed to the mild “punishment” during incorrect trials and to subsequently perform correction trials (CTs), i.e. the repetition of the same trial until touching correctly.

**Touchscreen Discrimination Training**

Discrimination training started on PND 106 ± 7 and was modified from Krakenberg and colleagues^1^ to further improve the learning performance of mice. Briefly, mice were trained to associate the positive cue position with the high-rewarded touch side and the negative cue position with the small-rewarded touch side. To achieve this aim, mice underwent 6 discrimination training steps which differed in regard to the maximum trial number, the learning criterion which had to be met to proceed to the following step, and the presence or absence of CTs. Furthermore, there were step-specific return-criteria, defining when mice had to be returned to a previous training step in case their performance was not adequate (supplementary table 1). In all steps, a session was terminated when the maximum number of trials was not reached after 30 min. Mice were always presented with positive and negative cues appearing in a pseudo-randomized order, so that the same cue was never presented more than four times in a row.

In discrimination training 1, the animals could always touch both symbols, but only the touch on the correct side led to the delivery of 7 µl SCM, while nothing happened when the incorrect cross was touched. In all following steps (discrimination trainings 2-6), correct touches in the positive condition led to the delivery of a large reward, while a small reward was presented in case of an incorrect touch. In the negative condition, correct touches led to a small reward, incorrect touches led to the mild “punishment”. In discrimination trainings 2 and 3, all incorrect touches were followed by CTs, i.e. the repeated presentation of the same cue until a correct touch was executed. In discrimination trainings 4, 5 and 6, CTs were presented only in the first part of the training session (for details see supplementary table 1). Furthermore, during these steps mice were presented with “pseudo-probe trials”. These were balanced numbers of positive and negative trials that resulted in a “neutral” outcome upon responding, meaning that they remained unpunished and/or unrewarded. The reason for introducing these trials was to accustom the mice to the later probe trials of the test which were also unpunished and unrewarded.

**Elevated plus-maze test**

The elevated plus maze test^4,5,6^ (EPM) was conducted on PND 245 ± 77. The apparatus was 50 cm elevated from the ground and consisted of four arms (30 × 5 cm² each) and a central square (5 × 5 cm²). There were two opposing open arms and two opposing closed arms, the latter ones being surrounded by 20 cm high walls. The open arms were only surrounded by a 4 mm high border to prevent the mice from falling off the maze. The floor of the maze was covered with a grey PVC inlay. The apparatus was illuminated by an LED lamp and the illumination level in the central square was 25 lux.

After spending 1 min in the transportation box in the test room, the test mouse was placed in the central square of the EPM, facing always the same closed arm. The mouse could then freely explore the apparatus for 5 min during which the experimenter was not present in the room.

The parameters analysed were the distance travelled on the open arms, the percentage of entries into the open arms (open arm entries / (open arm entries + closed arm entries) x 100), the percentage of time spent on the open arms (time on open arms / (time on open + time in closed arms) x 100) (anxiety-like behaviour) and the sum of entries into the open and closed arms (exploratory locomotion).

**Dark-light test**

The dark light test^7^ (DL) was performed on PND 247 ± 77. The apparatus consisted of a modified Makrolon cage type III, which was separated into two compartments. The dark compartment (14 x 26 x 15 cm³) was painted with black varnish and covered by a grey PVC lid. The light compartment (28 x 26 x 15 cm³) had translucent walls and was illuminated by an LED lamp, the illumination level was 40 lux. Both compartments were connected via a sliding door.

After transport to the testing room, each mouse spent 1 min in the dark compartment with the sliding door closed. Then the door was opened, and the mouse could freely explore the apparatus for 5 min during which the experimenter was not present in the room. The parameters analysed were the latency to enter the light compartment, the time spent in the light compartment (anxiety-like behaviour), and the number of entries into the light compartment (exploratory locomotion).

**Open field test**

The open field test8,9 (OF) was conducted on PND 252 ± 77. The apparatus consisted of a square plywood box (80 x 80 x 42 cm³) painted with white varnish. The central zone of the apparatus was defined as at least 20 cm distant from the walls. The arena was illuminated from above with an LED lamp, the illumination level was 35 lux in the centre. Each mouse spent 1 min in the transport box before being placed in always the same corner of the apparatus. The experimenter then left the room and the animal could freely explore the arena for 5 min. The parameters analysed were the time spent in the centre, the number of entries into the centre (anxiety-like behaviour), and the total distance travelled (exploratory locomotion).

**Supplementary table 1: Discrimination training steps as applied previously^1^ with modifications.** Discrimination training consisted of 6 steps. All sessions ended after maximally 30 min, unless the mouse reached the maximum number of trials before this time. During correction trials, animals were presented with the same condition until touching correctly. Pseudo-probe trials, i.e. balanced numbers of positive and negative trials that remained unpunished and/or unrewarded, were included to accustom the mice to the outcome of the probe trials during testing.

| Discrimination training | Max. number of trials | Learning criterion | Return criterion | Correction trials | Number of pseudo-probe trials |
| --- | --- | --- | --- | --- | --- |
| 1 | 50 | Minimally 5 days in this step, 50 trials in 20 min on 2 consecutive days | - | - | - |
| 2 | 20 | 80% correct responses and ≤ 7 CTs on two consecutive days | > 20 CTs or no CT reduction of 45% daily  🡪 discrimination training 1 | yes | - |
| 3 | 50 | 80% correct responses and ≤ 13 CTs on two consecutive days | > 30 CTs or no CT reduction of 45% daily  🡪 discrimination training 1 | yes | - |
| 4 | 50 | 80% correct responses and ≤8 CTs on two consecutive days | Learning criterion not met on 1 out of 4 days  🡪 discrimination training 3 | yes  (in trials 1-25) | 2  (pseudo- randomly distributed across trials 26-50) |
| 5 | 50 | 80% correct responses and ≤6 CTs on two consecutive days | Learning criterion not met on 1 out of 4 days  🡪 discrimination training 3 | yes  (in trials 1-15) | 4  (randomly distributed across trials 16-50) |
| 6 | 50 | 80% correct responses and ≤5 CTs on two consecutive days | Learning criterion not met on 1 out of 4 days  🡪 discrimination training 3 | yes  (in trials 1-5) | 6  (randomly distributed across trials 6-50) |

**Supplementary table 2: Results of pairwise comparisons of optimism scores in different conditions before and after experience phase.** Statistical information given: Holm-Bonferroni-corrected Wilcoxon signed rank test, V- and p-value. Sample size: n = 24. Bold: p < 0.001. Conditions: P = positive, NP = near positive, M = middle, NN = near negative, N = negative.

| **Before experience phase** | | | | | | | | | | |
| --- | --- | --- | --- | --- | --- | --- | --- | --- | --- | --- |
|  | P vs. NP | P vs. M | P vs. NN | P vs. N | NP vs. M | NP vs. NN | NP vs. N | M vs. NN | M vs. N | NN vs. N |
| **V** | 98 | 300 | 300 | 0 | 2.5 | 0 | 0 | 227 | 6 | 104 |
| **p** | 0.320 | **< 0.001** | **< 0.001** | **< 0.001** | **< 0.001** | **< 0.001** | **< 0.001** | **< 0.001** | **< 0.001** | 0.308 |
| **After experience phase** | | | | | | | | | | |
|  | P vs. NP | P vs. M | P vs. NN | P vs. N | NP vs. M | NP vs. NN | NP vs. N | M vs. NN | M vs. N | NN vs. N |
| **V** | 115 | 300 | 300 | 0 | 0 | 0 | 0 | 253 | 1 | 131 |
| **p** | 0.072 | **< 0.001** | **< 0.001** | **< 0.001** | **< 0.001** | **< 0.001** | **< 0.001** | **< 0.001** | **< 0.001** | 0.603 |


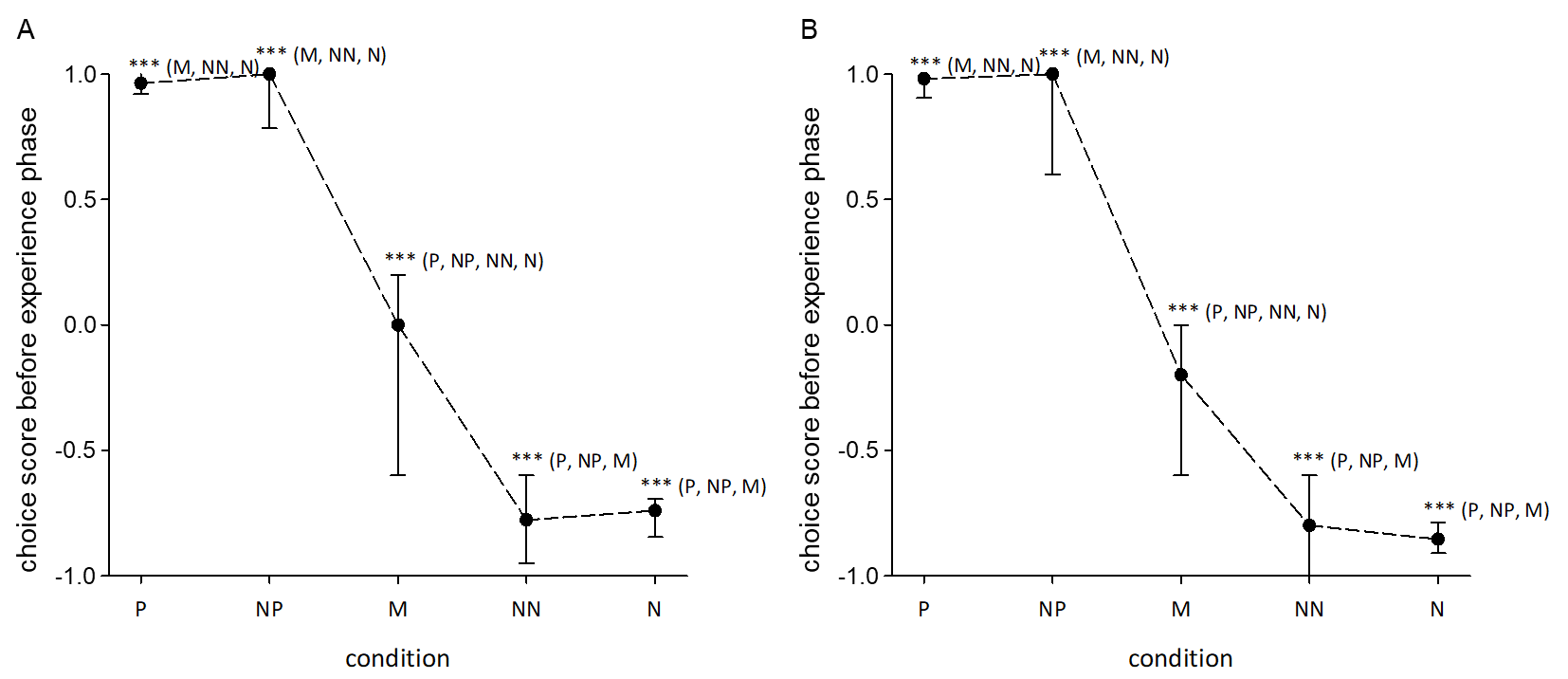


**Supplementary figure 1: Choice scores in response to the five conditions pooled across groups.** A: before experience phase, B: after experience phase. Data are presented as medians and 25^th^ and 75^th^ percentiles. Conditions: P = positive, NP = near positive, M = middle, NN = near negative, N = negative. Statistics: Holm-Bonferroni-corrected Wilcoxon signed rank test, n_A_ = n_B_ = 24, ***p ≤ 0.001.

**References**

[1] Krakenberg, V., Woigk, I., Garcia Rodriguez, L., Kästner, N., Kaiser, S., Sachser, N., et al. Technology or ecology? New tools to assess cognitive judgement bias in mice. Behavioural Brain Research, 362, 279–287 (2019). doi: 10.1016/j.bbr.2019.01.021

[2] Krakenberg, V., von Kortzfleisch, V. T., Kaiser, S., Sachser, N. & Richter, S. H. Differential effects of serotonin transporter genotype on anxiety-like behavior and cognitive judgment bias in mice. *Frontiers in Behavioral Neuroscience*, 13, 263 (2019). doi: 10.3389/fnbeh.2019.00263

[3] Richter, S. H., Vogel, A. S., Ueltzhöffer, K., Muzzillo, C., Vogt, M. A., Lankisch, K., et al. Touchscreen-paradigm for mice reveals cross-species evidence for an antagonistic relationship of cognitive flexibility and stability. *Frontiers in Behavioral Neuroscience*, 8, 154 (2014). doi: 10.3389/fnbeh.2014.00154

[4] Lister, R. G. The use of a plus-maze to measure anxiety in the mouse. *Psychopharmacology*, 92(2), 180–185 (1987).

[5] Lister, R. G. Ethologically-based animal models of anxiety disorders. *Pharmacology & Therapeutics*, 46(3), 321–340 (1990).

[6] Pellow, S., Chopin, P., File, S. E. & Briley, M. Validation of open : closed arm entries in an elevated plus-maze as a measure of anxiety in the rat. *Journal of Neuroscience Methods*, 14(3), 149–167 (1985).

[7] Crawley, J. N. & Goodwin, F. K. Preliminary report of a simple animal behavior model for the anxiolytic effects of benzodiazepines. *Pharmacology Biochemistry and Behavior*, 13(2), 167–170 (1980). doi: 10.1016/0091-3057(80)90067-2

[8] Archer, J. Tests for emotionality in rats and mice: a review. *Animal Behaviour*, 21(2), 205–235 (1973). doi: 10.1016/S0003-3472(73)80065-X

[9] Treit, D. & Fundytus, M. Thigmotaxis as a test for anxiolytic activity in rats. *Pharmacology Biochemistry and Behavior*, 31, 959–962 (1989)
